# Deep sequencing with longitudinal sampling of a VRC01-like-antibody response in a chronically infected individual

**DOI:** 10.1186/1742-4690-9-S2-O36

**Published:** 2012-09-13

**Authors:** Z Zhang, X Wu, N Longo, B Zhang, J Zhu, C NISC, JC Mullikin, L Wu, GJ Nabel, M Connors, PD Kwong, JR Mascola, L Shapiro

**Affiliations:** 1 Columbia University, New York, NY, USA; 2 Vaccine Research Center, NIAID, NIH, Bethesda, MD, USA; 3 National Human Genome Research Institute, Bethesda, MD, USA; 4 National Institute of Allergy and Infectious Diseases, NIH, Bethesda, MD, USA

## Background

VRC01-like antibodies use heavy chain mimicry of the CD4-receptor to achieve effective neutralization of HIV-1. The VRC01-like antibodies that have been observed in a number of HIV-1-infected individuals (i) display extensive somatic changes (70-100 nucleotide changes in VH-gene), (ii) can be detected only after several years of infection, (iii) derive from VH1-2, and (iv) are compatible with several different heavy J chains and different light chains.

## Methods

To understand the persistence, evolution, and lineage of VRC01-like antibodies, we sampled PBMCs from donor 45, the source of VRC01 and VRC03 antibodies, at approximately yearly intervals over a 15-year period, and performed deep sequencing on the heavy and light chain variable portions of expressed antibodies. Anti-idiotypic antibodies were used to correlate mRNA levels of antibodies identified by the deep sequencing with expressed levels of these antibodies in serum.

## Results

High expression levels of VRC01-like antibody sequences persisted over the entire 15-year period. Multiple lineages of VRC01-like antibodies were detected at each time point, and some of these, in particular the lineages that include VRC01 and VRC03, persisted over multiple time points, and displayed extensive branching in their evolution.

## Conclusion

Deep sequencing provides a means to define the genetic record of the lineage and maturation of antibodies effective at neutralizing HIV-1. Precise definition of the natural ontogeny of broadly neutralizing antibodies may be essential in defining appropriate strategies to elicit such antibodies in vaccine settings.

